# Determinants of stunting among Indonesian children: a cross-sectional study on the role of child, maternal, and vaccination factors

**DOI:** 10.1186/s12884-026-09373-x

**Published:** 2026-06-08

**Authors:** Mohammed Alfaqeeh, Neily Zakiyah, Maarten J. Postma, Auliya A. Suwantika

**Affiliations:** 1 Department of Pharmacology and Clinical Pharmacy, Faculty of Pharmacy, Universitas Padjadjaran, Bandung, 45363 Indonesia; 2 Center of Excellence for Pharmaceutical Care Innovation, Universitas Padjadjaran, Bandung, Indonesia; 3 Department of Health Sciences, University Medical Center Groningen, University of Groningen, Groningen, The Netherlands; 4 Department of Economics, Econometrics and Finance, Faculty of Economics and Business, University of Groningen, Groningen, The Netherlands; 5 Center for Health Technology Assessment, Universitas Padjadjaran, Bandung, Indonesia

**Keywords:** Birth weight, Children, Immunization, Indonesian Family Life Survey, Maternal nutrition, Risk factors, Stunting, Tetanus toxoid

## Abstract

**Background:**

Stunting remains a significant public health challenge in Indonesia, impacting children’s growth and development. This study aimed to identify child and maternal factors associated with stunting in children aged 0–59.

**Methods:**

This study employed a cross-sectional secondary data analysis using the Indonesia Family Life Survey-5 (2014). Variables were selected based on the United Nations International Children's Emergency Fund framework and prior evidence. Stunting was assessed using height-for-age z-scores (HAZ), with children classified as stunted if their HAZ was below -2 standard deviations. Descriptive statistics, bivariate analyses, and multivariable logistic regression were performed to explore associations between various factors and stunting, with adjusted odds ratios (aOR) and 95% confidence intervals (CI) calculated.

**Results:**

Of the 4,570 children included, 24.1% were classified as stunted. Factors that reduced the likelihood of stunting included older age, with children aged 12–23 months (aOR 0.111, CI 0.056–0.223) and 24–59 months (aOR 0.039, CI 0.021–0.075) at lower risk, likely due to improved dietary diversity and reduced vulnerability to early-life infections as children grow. Risk factors included low birth weight (< 2.5 kg, OR 3.556, CI 2.938–3.648) and incomplete immunization (aOR 1.511, CI 1.088–2.098). Maternal factors also played a role, with underweight mothers having a higher risk of stunting (aOR 2.122, CI 1.565–2.876), while overweight mothers (aOR 0.244, CI 0.105–0.570) and those who received tetanus toxoid injections (aOR 0.724, CI 0.561–0.935) had a lower risk.

**Conclusions:**

These findings highlight the critical role of age, birth weight, and vaccination status in the prevention of stunting among Indonesian children. Policies and programs should focus on improving maternal nutrition, ensuring full childhood immunization, and promoting appropriate complementary feeding practices during early infancy to help prevent stunting and improve child growth outcomes.

**Supplementary Information:**

The online version contains supplementary material available at 10.1186/s12884-026-09373-x.

## Introduction

Stunting is a condition characterized by impaired growth and development due to chronic malnutrition, and it remains a critical global health issue [1–3]. Stunting is primarily caused by poor nutrition during the first 1,000 days of life, which includes the period from conception to the child’s second birthday [4]. Children affected by stunting may never reach their full growth potential in height, and their cognitive development may also remain permanently hindered [5]. Moreover, children with stunted growth are at increased risk of lower educational achievement, and chronic diseases later in life [6], According to the World Health Organization (WHO), stunting is still the most predominant type of pediatric malnutrition that affects children globally [7].

Globally, stunting affects over 148 million children (22%) under five, with the burden concentrated in low- and middle-income countries (LMICs) [8]. Of these, about 83.8 million live in Asia and 58.7 million in Africa, highlighting persistent regional inequalities [9]. Indonesia, the most populous country in Southeast Asia [10], has one of the region’s highest stunting rates [11]. Although prevalence has gradually declined from around 37% in 2007 to about 28% in 2019 [12–14], progress remains slow. The National Health Insurance (JKN) program, introduced in 2014 to improve healthcare access and nutrition services, is particularly relevant to stunting prevention as it supports access to antenatal care, maternal nutrition counseling, childhood immunization, and routine growth monitoring services [15]. However, despite these improvements, stunting remains a major public health challenge in the country [16].

Multiple studies have highlighted that stunting is influenced by a combination of child-specific, economic, health, and maternal factors. Key demographic characteristics, such as a child's age and gender, affect growth patterns, with older children and boys being more susceptible [17–19]. Low birth weight also significantly increases the risk of stunting [20]. Nutritional factors are vital in preventing stunting, as early feeding practices like prelacteal food can disrupt exclusive breastfeeding and increase the risk of infections [21, 22], while exclusive breastfeeding for the first six months is recommended to protect against stunting [23]. Moreover, vaccination status is crucial, as complete immunization helps prevent infections that can lead to stunted growth [24].

Maternal and health service factors also substantially influence child nutritional status. These include maternal nutritional status, tetanus toxoid (TT) vaccination, health insurance coverage, and access to antenatal and postnatal care, all of which contribute to improved maternal and child health outcomes [25–28]. The use of the Mother and Child Health Book supports health knowledge, growth monitoring, and service utilization [29], while maternal education, employment status, and religious or cultural practices further shape feeding decisions and care behaviors [30–34]. These determinants operate through interrelated biological and environmental pathways. Inadequate maternal nutrition increases the likelihood of low birth weight, which in turn heightens vulnerability to infections during early childhood [35]. When combined with suboptimal feeding practices and incomplete immunization, these conditions can exacerbate growth faltering and increase the risk of chronic undernutrition [36]. Thus, maternal health, infant feeding behaviors, and access to preventive health services collectively shape child growth outcomes.

Although multiple studies have explored the determinants of stunting [37–40], most have examined socioeconomic disparity [41], vaccination [42], or maternal factors [43] independently, making it difficult to understand how these determinants interact to influence child growth. Prior research has shown that stunting arises from multi-level influences that operate simultaneously, including biological, caregiving, household, and environmental factors, and examining these determinants in isolation may underestimate their combined effect [44]. Furthermore, some studies focus on the first two years of a child’s life (0–2 years) [29] or 2–5 years [45, 46], overlooking a comprehensive assessment of stunting across the entire 0–5-year age range. This range represents the most critical window for linear growth, nutritional vulnerability, and responsiveness to prevention interventions, making it central for understanding stunting risks and informing early-life health strategies [47, 48].

Therefore, this study aims to address these gaps by jointly assessing childhood immunization coverage, various child-specific, and maternal factors in relation to stunting outcomes among children aged 0–5 years old using data from the Indonesian Family Life Survey (IFLS-5). IFLS-5 is particularly suited for this purpose because it provides linked maternal–child–household microdata along with detailed socioeconomic, health service utilization, and immunization variables, enabling integrated multivariable analysis of interacting risk pathways. In contrast, newer national surveys such as the Indonesian Nutritional Status Survey (*SSGI*), the Indonesia Health Survey (*SKI*), and the National Basic Health Survey (*Riskesdas*) are primarily designed for national prevalence monitoring and offer fewer behavioral, service-related, and contextual indicators, making them less suitable for investigating cross-factor interactions. While IFLS is longitudinal, a cross-sectional analytic approach is appropriate for identifying associations rather than modeling growth trajectories. By identifying key modifiable determinants, this study provides evidence that can support the design and prioritization of maternal nutrition programs, immunization strengthening strategies, and early child feeding interventions within Indonesia’s ongoing stunting reduction and primary healthcare policies.

## Methods

### Study design

This research employs a cross-sectional design utilizing secondary data from the IFLS-5 [49]. The survey collected extensive data on households, individuals, and communities using a multistage stratified sampling technique. Initially, the sample was drawn from 321 enumeration areas (EAs) across 13 of Indonesia's 27 provinces, with 20 households selected randomly from each urban EA and 30 households from each rural EA [50]. IFLS-5 provides a representative sample of 83% of the Indonesian population [51]. However, because the survey sample is concentrated primarily in western and central regions of Indonesia, particularly Java and Sumatra, findings should be interpreted with some caution when generalizing to underrepresented provinces in Eastern Indonesia. Additionally, several variables, including immunization history and maternal health information, were based on self-report, which may introduce recall or reporting bias. In IFLS-5, there was a transition in the data collection methodology, moving from traditional pen-and-paper personal interviews to computer-assisted personal interviews [52]. Additionally, anthropometric measurements for both children and parents were gathered by skilled interviewers as part of the data collection process in IFLS 5 [52]. It was also observed that the dynasty recontact rate stood at 92%, signifying the successful reestablishment of contact with families who had participated in previous waves of the survey. Moreover, the recontact rate for individual target households (including split-off households as separate entities) was noted to be 90.5% [51].

Inclusion criteria for this study consisted of children aged 0–59 months with available anthropometric measurements. Children outside this age range or those with missing height/age data required to calculate height-for-age z-score (HAZ) were excluded. This study utilized a total sampling approach, where all eligible cases meeting the inclusion criteria were included in the analysis. The dataset is freely accessible at https://www.rand.org/labor/FLS/IFLS.html. The study was funded by Survey METER and the RAND Corporation (US) [53]. Detailed information about the survey is available at https://www.rand.org/well-being/social-and-behavioural-policy/data/FLS/IFLS/ifls5.html

### Variables and measurements

Our analysis focused exclusively on the IFLS-5 cross-sectional survey data concerning children, defined as children aged 0 to 59 months [54]. The selection of variables for the multivariable regression model was guided by the United Nations International Children's Emergency Fund (UNICEF) conceptual framework for the determinants of child undernutrition, as well as evidence from previous empirical studies conducted in Indonesia and comparable LMIC settings [55–57]. This approach ensured that the included variables represented biologically and contextually relevant pathways influencing stunting. All included variables in this study were derived from the IFLS handbook, drawing from Books US, K, 3 A, 4, and 5. Full details of the included variables, including their definitions and response categories, are provided in the supplementary file (Table S1).

#### Stunting

The HAZ-score was used to measure stunting. HAZ-score is a standardized score used to assess stunting by comparing their height to age-specific reference populations [58]. According to the WHO standards [59], children with HAZ less than −2 standard deviations from the reference population median were classified as stunted more below. Within this classification, stunting is further categorized as moderate if the HAZ-score is between −2 and −3 SDs, and severe if the HAZ-score falls below −3 SDs. In this study, stunting was classified into two categories: stunted, where the child’s HAZ-score was more than two standard deviations below the median, and non-stunted, where the HAZ-score was equal to or above the median by more than two standard deviations. The measurements were obtained using the WHO Anthro software, which is designed to assess child growth based on WHO growth standards [60]. In the IFLS, physical health measurements, including anthropometrics, were conducted by professional nurses who accompanied the interviewers [61].

#### Vaccination

Indonesia implements a comprehensive routine childhood immunization program that includes primary and advanced vaccines scheduled from birth through the second year of life [62]. The primary schedule begins at birth with hepatitis B (HB), Bacillus Calmette–Guérin (BCG), and oral polio vaccine (OPV), followed by diphtheria–tetanus–pertussis–hepatitis B–Haemophilus influenzae type b (DTP–HB–Hib), OPV, and pneumococcal conjugate vaccine (PCV) at 2 and 3 months [62]. A repeat dose of DTP–HB–Hib, OPV, and inactivated polio vaccine (IPV) is given at 4 months, with measles–rubella (MR) administered at 9 months and a booster of DTP–HB–Hib and MR at 18–24 months [62].

For this study, complete childhood vaccination was operationalized as a composite binary variable. A child was categorized as “complete” if they had received all five core vaccines (BCG, OPV, DPT, Measles, and Hepatitis B). If at least one of these vaccines was not received, the child was classified as having “incomplete” vaccination. [62]. Vaccines such as PCV and IPV were not included in the completeness criteria because these vaccines had not yet been widely introduced into Indonesia’s national immunization program at the time IFLS-5 was conducted, and therefore coverage in the dataset was minimal and inconsistent across regions. Additionally, maternal immunization status was assessed by whether the mother reported receiving a TT injection during pregnancy.

#### Maternal factors

The study analyzed several maternal factors, including maternal education, occupation, ownership of a Mother and Child Health Book, antenatal and postnatal care visits, health insurance status, mother’s BMI, religion, and marital status. For analysis, maternal education was categorized into five levels (unschooled, elementary, junior high, senior high, and college), occupation was coded as employed or unemployed, and marital status was categorized as married or unmarried. Religion was grouped as Muslim or non-Muslim. Maternal BMI was categorized as underweight, normal, or overweight based on WHO classification. ANC and PNC visits, health book ownership, and health insurance use were each coded as yes/no variables. Full operational definitions and coding schemes are provided in Supplementary Table S1.

#### Child-specific factors

The factors assessed in children included age in months, gender, birth weight in kilograms, age of weaning in months, and age in months for the introduction of water and complementary feeding. Child age was categorized as 0–11 months, 12–23 months, and 24–59 months. Sex was classified as male or female. Birth weight was categorized into < 2.5 kg and ≥ 2.5 kg. The age at which water, complementary foods, and weaning were introduced was categorized as < 6 months or ≥ 6 months. Full definitions and coding for all child-specific variables are provided in Supplementary Table S1.

### Statistical analysis

Most of the included variables were grouped into two categories. These categories were consolidated to ensure adequate sample sizes within each group, allowing for meaningful analysis. Descriptive statistics were used to analyze the characteristics of the variables, while the chi-square test was employed to assess the univariate association between potential factors and the outcome. Subsequently, variables that showed an association with the outcome at a significance level of *p* < 0.25 were included in a multivariate logistic regression model. This *p*-value threshold is recommended because it allows for the consideration of variables that may have clinical significance, which could be overlooked if stricter thresholds like *p* < 0.05 are used [63]. To minimize potential confounding, relevant child and maternal variables identified from the UNICEF conceptual framework, prior literature, and bivariate screening were simultaneously included in the multivariable logistic regression model. We used the multivariable logistic regression to examine the associations between the assessed factors and stunting, with results reported as adjusted odds ratios (aOR) along with 95% confidence intervals (CI). The analysis was carried out using SPSS software (version 26, IBM Corporation, Armonk, NY, USA).

Missing data were handled using multiple imputation by chained equations (MICE) in SPSS software, with five imputed datasets generated [62]. This approach was used to reduce potential bias and retain the study sample size. All variables included in the final analysis were entered into the imputation model as both predictors and outcomes to improve estimation accuracy. Pooled estimates from the imputed datasets were used for the final regression analysis. Illustrations of missing data patterns are provided in the supplementary materials (Figure S1). To assess the quality of the imputations, density analysis was performed and showed that the distribution of imputed values closely aligned with the observed data (Figure S2).

## Results

### Baseline characteristics

**Fig. 1:**
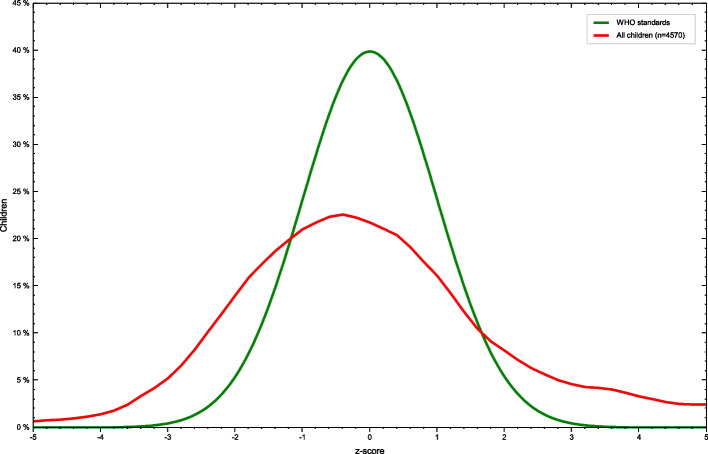
Prevalence of stunted children in our study compared to WHO reference values

Approximately half of the included children were female (52.7%), with the majority aged between 24–59 months (68.4%) and weighing more than 2.5 kg (91.6%). Most children were introduced to water at less than 6 months of age (75.1%) and started complementary food after 6 months (60.7%), while 79.8% were weaned after 6 months. Additionally, 67.6% of the children were fully immunized, and among all children, 24.1% were stunted. Regarding the mothers, around half were employed (49.2%), with a significant majority being married (94.5%) and having completed senior high school (39.5%). Most mothers identified as Muslim (87.8%), and a high percentage were underweighted (89.7%). Additionally, 89.5% had antenatal care, while 56.9% did not receive postnatal care. Furthermore, 71.2% received a TT injection, 61.4% had a Mother and Child Health Book, and 67.1% did not use health insurance. Full details are presented in Table 1, and the prevalence of stunted children in our study population compared to the reference values provided by the WHO is illustrated in Fig. 1.

**Table 1:** Characteristics of respondents (*n* = 4,570)

Variable	Categories	Presence of Stunting		*p*-value
		Yes N (%)	No N (%)	
Outcome variable				
Stunting		1103 (24.1)	3467 (75.9)	
Child variables				
Age	0–11	13 (0.3)	367 (8)	0.001**
	12–23	146 (3.2)	922 (20.2)	
	24–59	944 (20.7)	2178 (47.7)	
Sex	Male	696 (15.2)	1713 (37.5)	0.04*
	Female	608 (13.3)	1753 (38.4)	
Birth weight	Less than 2.5 kg	153 (3.3)	233 (5.1)	< 0.001**
	2.5 kg and more	950 (20.8)	3234 (70.8)	
Introduction of water	Less than 6 months	805 (16.7)	2668 (58.4)	< 0.004**
	More than 6 months	279 (6.1)	861 (18.8)	
Introduction of complementary food	Less than 6 months	418 (9.1)	1380 (30.2)	0.151
	More than 6 months	685 (15)	2087 (45.7)	
Weaning age	Less than 6 months	157 (3.4)	766 (16.8)	< 0.001**
	More than 6 months	946 (20.7)	2701 (59.1)	
Vaccination	Not fully immunized	349 (7.6)	1130 (24.7)	0.161
	Fully-immunized	755 (16.5)	2337 (51.1)	
Maternal variables:				
Marital status	Married	1046 (22.9)	3308 (71.6)	0.199
	Unmarried	57 (1.2)	159 (3.5)	
Education	Elementary	219 (4.8)	677 (14.8)	< 0.001**
	Junior high	388 (8.5)	661 (14.5)	
	Senior high	353 (7.7)	1454 (31.8)	
	College	143 (3.1)	675 (14.8)	
Occupation	Employed	559 (12.2)	1690 (37)	< 0.001**
	Unemployed	544 (11.9)	1777 (38.9)	
Religion	Muslim	986 (21.6)	3026 (66.2)	< 0.001**
	Non-Muslim	118 (2.6)	440 (9.6)	
Body max index	Underweight	952 (20.8)	3151 (68.9)	< 0.001**
	Normal	286 (6.3)	120 (2.6)	
	Overweight	31 (0.7)	30 (0.7)	
Antenatal care	Yes	965 (21.1)	3125 (68.4)	0.397
	No	138 (3)	342 (7.5)	
Postnatal care	Yes	457 (10)	1512 (33.1)	0.442
	No	646 (14.1)	1955 (42.8)	
Tetanus Toxoid injection	Yes	836 (18.3)	2417 (52.9)	< 0.001**
	No	268 (5.9)	1050 (23)	
Mother and Child Health Book	Yes	649 (14.2)	2159 (47.2)	0.24
	No	454 (9.9)	1308 (28.6)	
Health insurance	Yes	360 (7.9)	1147 (25.1)	0.105
	No	743 (16.3)	2320 (50.8)	

*Significant level 0.05, **Significant level 0.01

### Association between potential factors and stunting

Bivariate analysis is an essential tool for understanding the associations between different variables. Our analysis revealed several factors that are statistically significantly associated with stunting, indicated by *p*-values of less than 0.05. For the child, significant variables include age, sex, birth weight, introduction of water, and age of weaning. For the mother, significant variables include education, occupation, religion, BMI, and receipt of the TT injection. Conversely, the child-related variables that did not show statistically significant associations with stunting are the introduction of complementary food (*p* = 0.151) and vaccination status (p = 0.161), while the mother-related non-significant variables are marital status (*p* = 0.199), postnatal care (*p* = 0.442), ownership of the Mother and Child Health Book (*p* = 0.240), and health insurance (*p* = 0.105).

In the multivariable logistic regression analysis, child factors showed significant associations with stunting. Children aged 12–23 months had an aOR of 0.111 (CI: 0.056–0.223) and those aged 24–59 months had an aOR of 0.039 (CI: 0.021–0.075), both indicating a strong protective effect against stunting compared to children aged 0–11 months. Birth weight also significantly affected stunting, with children weighing less than 2.5 kg having an aOR of 3.556 (CI: 2.938–3.648), compared to those weighing 2.5 kg or more, indicating that lower birth weight is strongly associated with an increased risk of stunting in children. Furthermore, children who were not fully immunized had a higher risk of stunting, with an aOR of 1.511 (CI: 1.088–2.098) (*p* = 0.019). Other factors, including sex, introduction of water, complementary food timing, and weaning age, did not show significant associations.

Maternal factors also played a significant role. Underweight mothers were at a higher risk of having stunted children (aOR: 2.122, CI: 1.565–2.876), overweight mothers showed a protective effect (aOR: 0.244, CI: 0.105–0.570). Mothers who received TT injections had a lower risk of having stunted children (aOR: 0.724, CI: 0.561–0.935) (*p* = 0.016). Other maternal factors, including marital status, education, employment, religion, having a Mother and Child Health Book, and health insurance, were not significantly associated with stunting. Further details are presented in Table 2.

**Table 2:** Multivariable regression analysis of study variables associated with stunting

Variables	Categories	Adjusted OR	95% CI	*P* value
Child variables				
Age	12–23	0.111	0.056–0.223	0.001**
	24—59	0.039	0.021–0.075	
	0–11	Reference		
Sex	Male	0.831	0.608–1.136	0.211
	Female	Reference		
Birth weight	Less than 2.5 kg	3.556	2.938–3.648	0.001**
	2.5 kg and more	Reference		
Introduction of water	Less than 6 months	1.257	0.954–1.657	0.099
	More than 6 months	Reference		
Introduction of complementary food	Less than 6 months	0.818	0.513—1.305	0.336
	More than 6 months	Reference		
Weaning age	Less than 6 months	1.934	0.702–5.328	0.153
	More than 6 months	Reference		
Vaccination	Not fully immunized	1.511	1.088–2.098	0.019*
	Fully-immunized	Reference		
Maternal variables:				
Marital status	Married	1.160	0.704–1.911	0.541
	Unmarried	Reference		
Education	Elementary	0.672	0.117–3.859	0.571
	Junior high	0.277	0.066–1.162	0.069
	Senior high	0.795	0.367–1.722	0.490
	College	Reference		
Occupation	Employed	1.124	0.431–2.923	0.759
	Unemployed	Reference		
Religion	Muslim	0.694	0.139–3.476	0.574
	Non-Muslim	Reference		
Body max index	Underweight	2.122	1.565–2.876	0.001**
	Overweight	0.244	0.105–0.570	
	Normal	Reference		
Tetanus Toxoid injection	Yes	0.724	0.561–0.935	0.016*
	No	Reference		
Mother and Child Health Book	Yes	1.036	0.579–1.857	0.668
	No	Reference		
Health insurance	Yes	1.036	0.579–1.857	0.882
	No	Reference		

*Abbreviations*: *OR* Odds ratio, *CI* Confidence interval

*Significant level 0.05 **Significant level 0.01, A multivariable logistic regression model was used

## Discussion

Of all the children included in the study, nearly a quarter were stunted. Age, birth weight, and vaccination status were significantly associated with stunting, with older age and higher birth weight showing a protective effect, while incomplete immunization increased the risk. Maternal factors such as being underweight were associated with a higher risk of stunting, while receiving tetanus toxoid injections and being overweight were linked to reduced risk.

Among child factors, our study found that children aged 12–23 months and 24–59 months were significantly less likely to be stunted compared to children aged 0–11 months. This finding is consistent with another study conducted in Indonesia using a different data source, which reported that younger children, those aged below 12 months, were more likely to be at risk of stunting [64]. However, other studies conducted in Nepal and Nigeria have reported that older children, particularly those aged 24–59 months, have a higher likelihood of being stunted compared to younger age groups, indicating a gradual increase in stunting risk as children age [65, 66]. Children under 12 months are more vulnerable to stunting due to several key factors. This period is critical for growth, and inadequate nutrition, such as poor breastfeeding.

practices and the early introduction of inappropriate complementary foods can impact development [67, 68]. A study found that young infants are more susceptible to infections like diarrhea and respiratory illnesses, which impair nutrient absorption and contribute to malnutrition [69]. These differing findings suggest that the association between age and stunting may vary across settings and should be interpreted cautiously. In our cross-sectional study, the lower odds observed among older children may reflect cohort differences, selective survival, or partial catch-up growth after infancy rather than a direct protective effect of age.

Birth weight also played a significant role in stunting. In line with our findings, children born weighing less than 2.5 kg having over three times the risk of stunting compared to those with a higher birth weight. This aligns with numerous studies, especially with those from LMICs, which consistently report that low birth weight is a critical risk factor for poor growth outcomes in early childhood [70–72]. Children with low birth weight often face challenges in both short- and long-term growth due to factors like compromised nutrition during the prenatal period. A study found a significant association between maternal nutritional status during pregnancy, iron intake, and gestational age with the occurrence of low birth weight [73]. Another study highlighted that inadequate fetal growth or stunting within the first two years of life can cause long-term effects, including shorter adult stature, lower levels of educational attainment, reduced income in adulthood, and lower birth weight in the next generation [74].

Vaccination status emerged as another important determinant of stunting. In line with our finding, many studies reported that incomplete vaccination was significantly associated with stunting, with children who had not received all their vaccinations being at a higher risk of stunting compared to those who were fully immunized [24, 75–77]. One possible explanation for the association between immunization status and stunting is that vaccines protect children from infectious diseases that contribute to malnutrition and impaired growth [78]. By preventing illness, vaccinations reduce the likelihood of secondary infections, which can worsen malnutrition and increase the risk of stunting. Therefore, full immunization supports a cycle of improved health and growth in children [79]. Many vaccination programs also integrate nutrition education and maternal-child health services, which further support child health and development, including growth outcomes[80]. Through these mechanisms, immunization can play a significant role in promoting healthy child growth. New vaccinations may indirectly support better nutritional status and growth outcomes in children, as they play a crucial role in preventing infectious diseases that are particularly harmful to children under five. For example, vaccines targeting diseases such as rotavirus and pneumococcal infections can help reduce the incidence of diarrhea and respiratory illnesses, which are known to contribute to malnutrition and impaired growth [81, 82].

Our study also found that mothers who received TT vaccine had a reduced risk of their children being stunted, which aligns with findings from a study conducted in the Philippines. In that study, the TT vaccination was associated with a lower likelihood of stunting, with an OR of 0.67 (*p* = 0.011), suggesting that maternal vaccination plays a crucial role in reducing stunting risk by improving both maternal and child health outcomes [83]. This protective effect is likely due to the overall improvement in health and immune responses that vaccination provides during pregnancy [84]. A study explained that maternal antibodies transferred both trans-placentally and through breast milk play a critical role in protecting neonates from infectious diseases during the first few weeks of life, a period marked by their vulnerability [85]. Therefore, providing passive immunity, maternal antibodies help prevent such infections, thereby reducing the risk of stunting. Additionally, vaccinations against other infections, such as pertussis and influenza, are also essential. Maternal immunization against pertussis, for instance, not only protects mothers but also provides critical protection to newborns, thereby further contributing to improved growth outcomes [86].

Among maternal factors, our study reported that maternal BMI was a significant predictor of stunting. Mothers who were underweight had more than twice the risk of having stunted children, while those who were overweight had a lower risk. A study mentioned that women with underweight BMI had 6% higher odds of having stunted children compared to those with normal BMI [72]. One possible explanation drawn from Tanzanian research is that maternal micronutrient supplementation during pregnancy not only increased gestational age and improved birth weight but also enhanced intrauterine growth[87]. These improvements were particularly important for reducing stunting risks, especially among underweight mothers. A Nigerian study suggested that interventions focusing on better nutrition, and child feeding practices have been highlighted as essential strategies for lowering the risks associated with maternal underweight status [88]. On the other hand, our study reported that overweight mothers have a reduced risk of stunting in their children. A similar study using the same data source found that obese mothers had a lower risk of having stunted children, though they classified BMI into four categories normal, underweight, overweight, and obese, while our study grouped BMI into only three categories underweight, normal, and overweight [45]. It should be noted that the term ‘overweight' in this study refers to mothers with a BMI in both the overweight and obese categories. While this grouping simplifies the analysis, it may overlook differences in health risks associated with obesity. Despite differences in classification, the underlying protective effect observed may relate to maternal nutritional status. This may be supported by evidence suggesting that maternal overnutrition can provide sufficient energy and nutrient reserves during pregnancy, positively influencing fetal growth [89]. However, excess maternal weight has also been associated with complications such as gestational diabetes and hypertension, which can impact placental function and nutrient delivery to the fetus [90, 91]. While some studies show that these conditions could hinder fetal growth, they may also, in other contexts, lead to higher birth weights, which are sometimes protective against stunting [92].

In this study, several variables did not show a statistically significant association with stunting, including child sex, timing of water and complementary food introduction, weaning age, maternal marital status, education level, occupation, religion, use of the Mother and Child Health Book, and health insurance coverage. The lack of association for early feeding practices may be explained by variability in how these behaviors are implemented and recalled, as well as potential reporting bias among caregivers [93]. Additionally, maternal education, marital status, and occupation may exert indirect effects on child nutrition through household income or caregiving support, which were not directly measured in the present model [94, 95]. The non-significant association for health insurance and use of the Mother and Child Health Book suggests that access alone may not be sufficient without consistent utilization and quality of counseling [96]. Together, these findings indicate that the determinants of stunting are multifactorial, and some influences may operate through broader socioeconomic and environmental pathways rather than direct individual-level behaviors.

This study presents several strengths and limitations that are important to consider. A notable strength is the sample size of 4,750 respondents, which enhances the statistical power and generalizability of the findings across the Indonesian population. Additionally, the study employed rigorous data imputation techniques, minimizing the potential bias associated with missing data and improving the reliability of the results. Furthermore, the analysis conducted in this study incorporated a wide range of potential risk factors to comprehensively address the various variables that may be associated with stunting. However, limitations include the reliance on self-reported data, which can introduce recall bias. Furthermore, while the cross-sectional design provides valuable insights, it does not establish causality between the factors and stunting. Furthermore, the geographic distribution of IFLS-5 may underrepresent some eastern provinces, and results may not fully capture regional variation in stunting patterns. The study may also be limited by the exclusion of certain sociodemographic variables that could influence stunting, such as socioeconomic status and access to healthcare. Additionally, Additionally, the term 'overweight' combines both overweight and obese categories, which may overlook distinct health risks, and future studies should consider differentiating between these groups to better understand their specific health impacts. Addressing these limitations in future research could further strengthen our understanding of stunting in Indonesia.

There is an urgent need to strengthen public health strategies to reduce stunting in Indonesia. Enhancing maternal nutrition before and during pregnancy should be prioritized, particularly in regions with high rates of undernutrition, through improved antenatal care and routine provision of micronutrient supplementation [97]. Social protection programs that provide food assistance and nutrition education for women of reproductive age are also essential, especially in rural and low-income communities [98]. Strengthening childhood and maternal TT immunization efforts can further reduce infection-related growth impairment, and expanding vaccination outreach through community health posts (Posyandu), primary health centers (Puskesmas), and mobile health services can help ensure equitable access [99]. Tailored interventions are needed for infants aged 0–11 months—the age group at highest risk of stunting—with a focus on timely initiation of complementary feeding and age-appropriate weaning practices supported by community-based counseling programs [100]. Community pharmacists can also contribute by counseling on micronutrient supplementation and supporting maternal and child vaccination awareness [101]. Finally, improving the capacity of frontline health workers, including midwives, nutritionists, and community health volunteers, is crucial to ensure early identification and management of stunting risk factors at the community level [102].

## Conclusion

Stunting remains a critical public health issue among Indonesian children, with consequences for physical growth, cognitive development, and long-term well-being. This study identified protective factors such as older child age, adequate birth weight, and complete immunization, while low maternal BMI and insufficient tetanus toxoid vaccination increased the likelihood of stunting. These findings highlight the need to strengthen maternal health and early childhood nutrition across existing national health platforms, including *Posyandu* and *Puskesmas* for routine growth monitoring, breastfeeding counseling, and complementary feeding support, as well as the JKN program to improve access to antenatal and postnatal care. Alignment with the National Strategy for Stunting Reduction (Stranas Stunting) can help ensure multi-sector coordination and program consistency. Priority interventions should include maternal nutrition supplementation, support for exclusive breastfeeding, promotion of diverse complementary feeding practices, and ensuring complete childhood immunization coverage. In the long term, reducing stunting is essential for improving educational achievement, productivity, and the overall quality of Indonesia’s human resources, making early-life nutrition and maternal health investments not only a health priority but also a strategic national development imperative.

## Supplementary Information

Supplementary Material 1.

## Data Availability

The dataset for this study is openly available from the IFLS, a joint initiative by RAND Corporation, Gadjah Mada University, and Survey METER. It can be accessed via the following link: [https://www.rand.org/labor/FLS/IFLS.html] (https:/www.rand.org/labor/FLS/IFLS.html).

## Abbreviations

WHO: World Health Organization
SEA: Southern and Southeastern Asia
JKN: National Health Insurance
HAZ: Height-for-age z-scores
LMICs: Low- and middle-income countries
IFLS: Indonesian Family Life Survey
BMI: Body mass index
EAs: Enumeration areas
BCG: Bacille Calmette-Guérin
OPV: Oral Polio Vaccine
DPT: Diphtheria, Pertussis, and Tetanus
HB: Hepatitis B
TT: Tetanus Toxoid
UNICEF: United Nations International Children's Emergency Fund
