# Supplementary material for: Determinants of stunting among Indonesian children: a cross-sectional study on the role of child, maternal, and vaccination factors
Source: BMC Pregnancy Childbirth. 2026 Jun 8;26:884. doi: 10.1186/s12884-026-09373-x (PMC13463939; doi:10.1186/s12884-026-09373-x)
Supplement: Supplementary file 1 [file 12884_2026_9373_MOESM1_ESM.pdf]

SUPPLEMENTARY FILE

Table S1. Variable Definitions and Response Categories

Variable	Definition	Response categories
**Outcome variable**		
**Stunting**	Child's height (in cm) being significantly below the norm for their age (in months)	1 = Stunted<br>2 = Non-Stunted
**Child variables**		
**Age**	Child's age	1 = 12-23<br>2 = 24-59<br>3 = 0-11
**Sex**	Child's sex	1 = Male<br>2 = Female
**Birth weight**	Child's Birth weight (in kg)	1 = Less than 2.5 kg<br>2 = 2.5 kg and more
**Introduction of water**	Child's age when first fed water (in months)	1 = Less than 6 months<br>2 = More than 6 months
**Introduction of complementary food**	Child's age when first fed other foods (in months)	1 = Less than 6 months<br>2 = More than 6 months
**Weaning age**	Child's age when first weaned (in months)	1 = Less than 6 months<br>2 = More than 6 months
**Vaccination**	Receipt of Bacillus Calmette-Guérin (BCG), Oral Polio Vaccine (OPV), Diphtheria-Pertussis-Tetanus (DPT), Measles, and Hepatitis B (HB) by the child	1 = Not fully immunized<br>2 = Fully immunized
**Maternal variables:**		
**Marital status**	Mother's marital status	1= Married<br>2= Unmarried
**Education**	Mother's highest level of attained education	1 = Unschooled<br>2 = Elementary<br>3 = High Junior<br>4 = Senior High<br>5 = College
**Occupation**	Whether the mother is working or not	1 = Yes<br>2= No
**Religion**	Mother's religion	1 = Muslim<br>2= Non-Muslim

**Body max index**	Mother's body max index	1 = Underweight<br>2 = Overweight<br>3 = Normal
**Antenatal care**	Mother's check-up by skilled health professional during pregnancy	1 = Yes<br>2= No
**Postnatal care**	Mother's follow-up by the healthcare provider who delivered the baby in the first 40 days after birth	1 = Yes<br>2= No
**Tetanus Toxoid injection**	Whether the mother received Tetanus Toxoid (TT) vaccine during pregnancy	1 = Yes<br>2= No
**Mother and Child Health Book**	The ownership of Mother and Child Health Book	1 = Yes<br>2= No
**Health insurance**	The use of health insurance to pay for all or some of this visit	1 = Yes<br>2= No

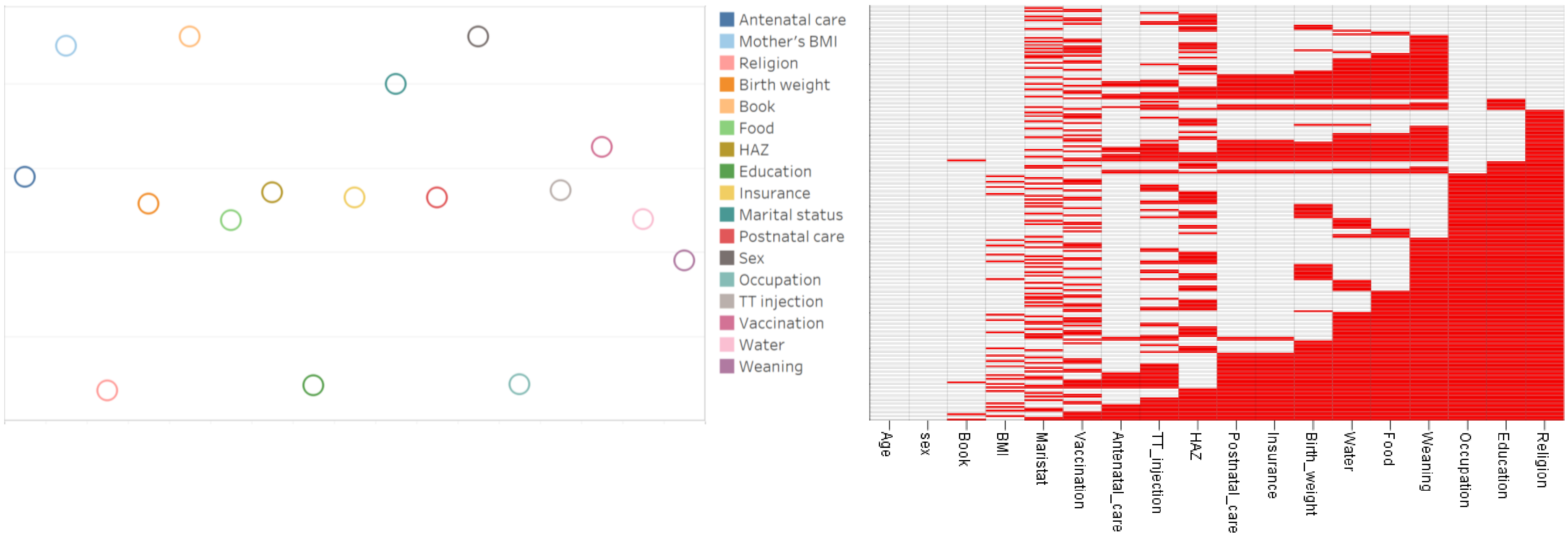

Figure S1: Circle view visualization and the pattern of missing data

Notes: BMI (Mother's body max index); HAZ (Stunting); education (Mother's Education); Age (Child's Age); Antenatal Care (Mother's Antenatal care), Postnatal Care (Mother's Postnatal care); Water (Child's Age for Introduction of Water); Religion (Mother's Religion); Book (Mother and Child Health Book); Food (Child's Age for Introduction of Complementary Food); Insurance (Mother's Health Insurance); Birth Weight (Child's Birth Weight); Weaning (Child's Weaning Age); Vaccination (Child's Vaccination); TT Injection (Mother's Tetanus Toxoid injection); Sex (Child's Sex); Maristat(Mother's Marital Status); Occupation(Mother's Occupation).

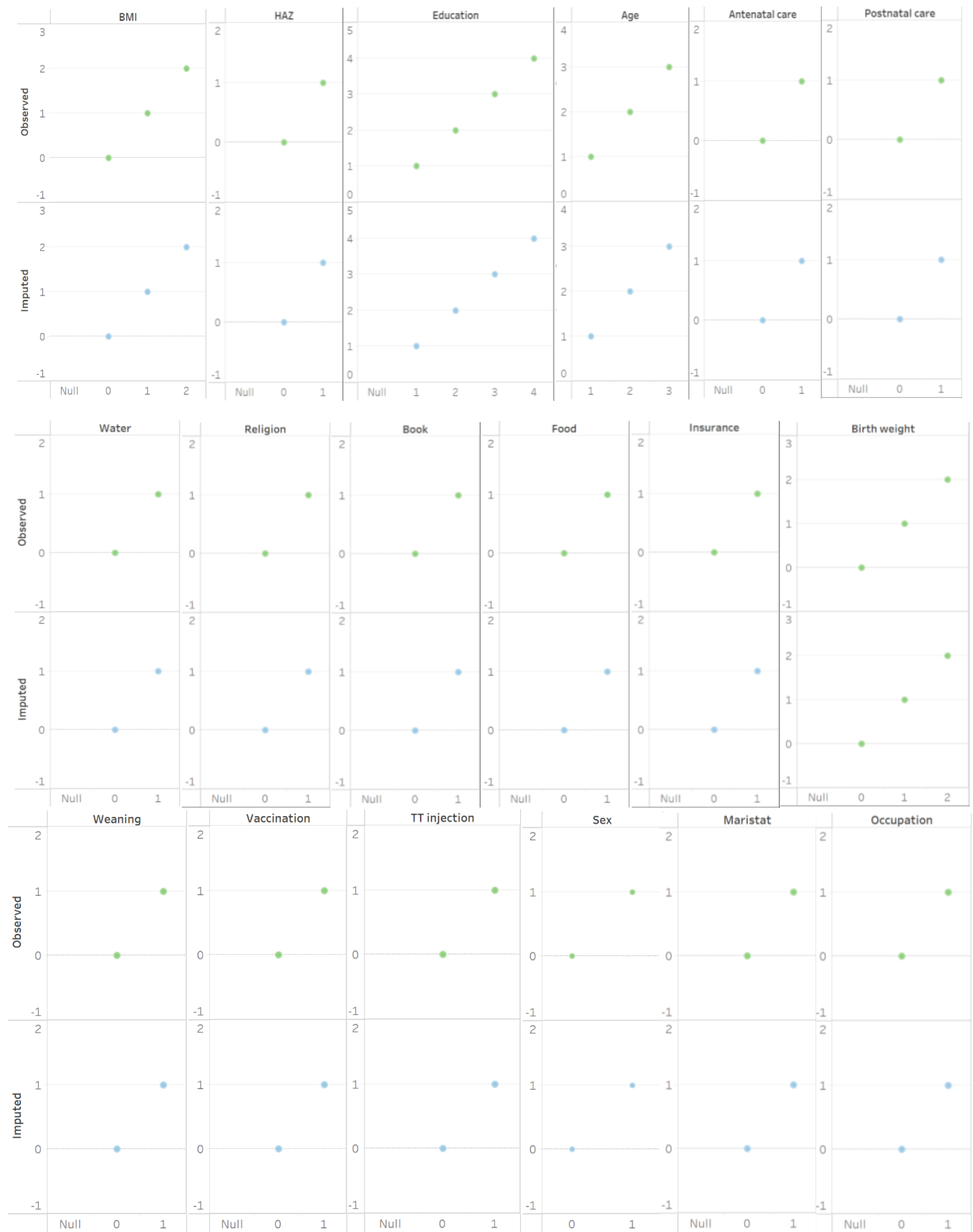

Figure S2: The density analysis of original (observed) and imputed data

Notes: BMI (Mother's body max index); HAZ (Stunting); education (Mother's Education); Age (Child's Age); Antenatal Care (Mother's Antenatal care), Postnatal Care (Mother's Postnatal care); Water (Child's Age for Introduction of Water); Religion (Mother's Religion); Book (Mother and Child Health Book); Food (Child's Age for Introduction of Complementary Food); Insurance (Mother's Health Insurance); Birth Weight (Child's Birth Weight); Weaning (Child's Weaning Age); Vaccination (Child's Vaccination); TT Injection (Mother's Tetanus Toxoid injection); Sex (Child's Sex); Maristat(Mother's Marital Status); Occupation(Mother's Occupation).
